# Multicomponent Exercise Training Improves Gait Ability of Older Women Rather than Strength Training: A Randomized Controlled Trial

**DOI:** 10.1155/2020/6345753

**Published:** 2020-09-16

**Authors:** Renata Wolf, Rafaella R. Locks, Paula B. Lopes, Paulo C. B. Bento, André L. F. Rodacki, Attilio N. Carraro, Gleber Pereira

**Affiliations:** ^1^Department of Physical Education, Federal University of Parana, Curitiba, Paraná, Brazil; ^2^Faculty of Education, Free University of Bozen-Bolzano, Brixen-Bressanone, Italy

## Abstract

**Objective:**

The aim of this study was to compare the effects of multicomponent and strength training programs on dynamic balance, functional capacity, and gait ability in older women.

**Methods:**

Thirty individuals (67 ± 4.3 years; 30.6 ± 3.9 kg/m^2^) were trained for 12 weeks (3 times per week), following multicomponent (MG: exercises focusing on agility, balance, muscle strength, and aerobic) and strength programs (SG: lower limbs strength exercise).

**Results:**

Peak torque of hip flexors (*p*=0.020) and extensors (*p*=0.009) and knee flexors (*p*=0.001) of SG was greater than that of MG at posttraining. In addition, both groups increased peak torque of knee extensors (*p*=0.002) and plantar extensors with higher effect size for SG (*d* = −0.41 and −0.48), whereas MG presented higher effect size for plantar flexors muscles (*d* = −0.55). Only the SG improved the rate of torque development of knee extensors (29%; *p*=0.002), and this variable was also greater to SG than MG at posttraining (106%). The SG and MG improved dynamic balance although SG presented higher effect size (*d* = 0.61). Both groups improved the performance on 30 s sit to stand test (*p*=0.010) with higher effect size for MG (*d* = −0.54). Only the MG improved the stride length (4%; *p*=0.011) and gait speed (10%; *p*=0.024). In addition, the groups improved toe clearance (*p*=0.035) and heel contact (*p*=0.010) with higher effect sizes for MG (*d* = −0.066 and 1.07).

**Conclusion:**

Strength training should be considered to increase muscle function and dynamic balance in older women, whereas multicomponent training should be considered to increase functional capacity and gait ability in this population.

## 1. Introduction

Exercise training is recommended to reduce the risk of many adverse health outcomes [1], such as dependence in performing the activities of daily living [2]. Such dependence is related to the decline in strength and in the capacity of producing force rapidly [3], resulting in the decline in the dynamic balance, in the walking ability, and in the functional capacity [4]. Therefore, exercise training program for older adults should include aerobic, muscle strengthening, balance, and walking exercises [5] to reverse or minimize these aging effects on muscle function as well as on functional capacity.

Improvements in muscle strength, rate of torque development (RTD), and gait are shown after strength training programs [6–8]. The adaptive changes in muscle functioning after strength training (e.g., firing frequency and recruitment of motor units) may be responsible to improve these parameters [9]. However, such adaptive changes may not improve functional capacity [6] due to the low mechanical specificity between strength training exercises and daily-life activities [2, 10]. Thus, the multicomponent training program has been used in an attempt to enhance the performance in daily-life activities of older adults.

Multicomponent training programs are characterized by periods in which several motor abilities are emphasized (e.g., strength, balance, and gait ability) [2]. It has been suggested that these programs are effective in improving older adults' functional capacity because the physical exercise movements mimic the activities of daily living (e.g., walking and squatting). Moreover, there is evidence that multicomponent training is effective to improve muscle strength of the ankle muscles that are related to the walking ability [11].

Indeed, some studies have reported gait speed improvements after a multicomponent training period [11] using indirect assessments (e.g., timed up and go and 10-meter walk tests) [12] and observational scales [13]. However, direct parameters of gait ability (e.g., toe clearance) are as important as gait speed to maintain the independence in daily living activities and avoid falls. Thus, a more comprehensive analysis is required to determine if multicomponent training can produce changes in older adults' gait ability. Furthermore, there is no direct comparison between multicomponent and strength training on effects of gait ability. Therefore, the aim of this study was to compare the effects of multicomponent and strength training programs on the muscle strength, dynamic balance, functional capacity, and gait ability in older women.

## 2. Methods

### 2.1. Participants

**Figure 1:**
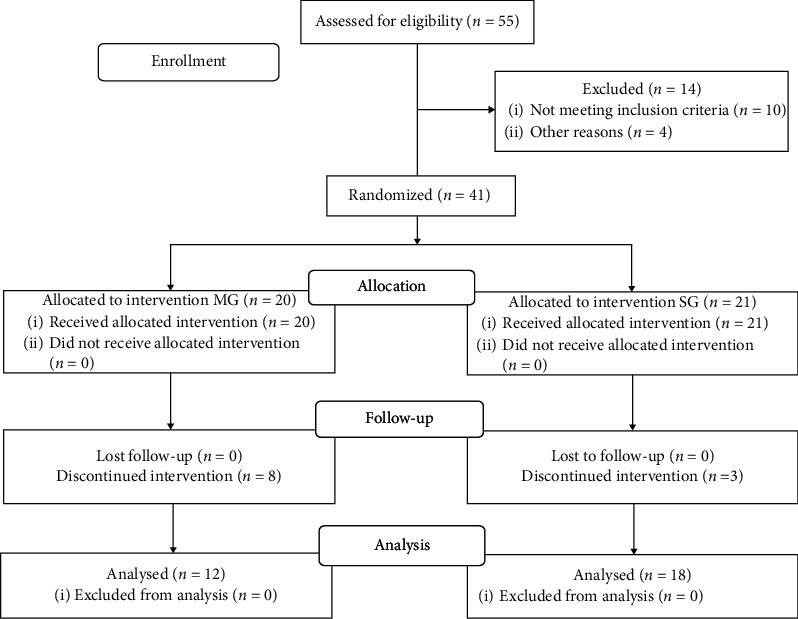
Consort flow diagram. MG: multicomponent group; SG: strength group.

The older women were invited to participate in this research through local newspapers and flyers distributed in churches, community centers, and health units in the city of Curitiba, Parana, Brazil. Eighty-two older women contacted the researchers. The inclusion/exclusion criteria to participate in the study were being able to walk without device, performing daily tasks independently, no participation in systematized exercise programs six months prior this study, and absence of cognitive deficits that could interfere on the comprehension of the exercises. Fifty-five older women were assessed for eligibility, and after applying the inclusion/exclusion criteria, forty-one older women gave their written consent to participate in the study and were randomly assigned to one of the following two groups: multicomponent (MG) and strength (SG). Eleven participants discontinued intervention due to personal reasons. The data of 30 participants (MG *n* = 12; 68 ± 3.4 years; 30.9 ± 4.3 kg/m^2^; SG: *n* = 18; 67 ± 6 years; 30.1 ± 3.3 kg/m^2^) were assessed (Figure 1).

### 2.2. Experimental Design

Maximal voluntary isometric contraction (peak torque and rate of torque development of hip, knee, and ankle extensors and flexors muscles), postural control (voluntary step execution test [14]), functional capacity (6 min walking test, sit and reach, 8-ft up and go, and 30 s chair stand tests), and gait ability (stride length, gait cycle duration, cadence, gait speed, toe clearance, heel contact speed, swing duration, stance duration, and double support duration) were assessed before (pre) and after (post) 12 weeks of exercise training. The evaluations were performed by the same two experienced researchers blinded from the allocation group. Pretraining assessments were performed during three days in the week preceding the training period. The first test session consisted of the dynamic balance test and familiarization with maximal voluntary isometric test. The second session was used to determine the functional capacity and the maximal voluntary isometric contraction torque and the third one to perform gait analysis. The groups had been reassessed 72h after the last training session, using the same procedures of the pretest evaluation. The local Ethics Committee approved the experimental procedures (1091.016.11.03). The study was registered in the Brazilian Registry of Clinical Trials (RBR-8mqt4m).

### 2.3. Maximal Voluntary Isometric Contraction

Hip, knee, and ankle extensor and flexor torques were assessed in a recumbent posture, having proximal segments firmly secured and stabilized by a Velcro strap, and the tested segments were positioned at approximately 90° [6, 8]. The tests of the torque joint followed a reversed balanced order. Force-time traces were obtained using a load cell (Model CZC500, Kratos, São Paulo, Brazil) and sampled at 1 kHz, and data were converted to digital signals with the aid of a 16 bit A/*D* card (National Instruments, Model NI USB 6218, USA) and stored on a personal computer. Raw torque data were low-pass filtered (20 Hz) with a Butterworth 4th order recursive filter. The load cell was firmly attached to an adjustable pole, perpendicularly aligned with the dominant (tested) segment. The perpendicular distance between the load cell and the estimated joint center of rotation was determined and used to calculate net joint torques. The participants were instructed to produce torque as fast and hard as possible and to sustain the contraction for approximately 2-3 s. One-minute rest interval was set among three attempts. The highest peak torque obtained was used for further analysis. Peak torque was determined as the highest torque value obtained after the onset of the voluntary contraction. The rate of torque development was defined as the slope of the force-time curve from 20% to 80% of the peak torque values [15]. The coefficient of determination was calculated to assess the fitting of the regression equations (*R*^2^ = 0.98). Both variables were calculated using a customized routine (Matlab 7.0, USA).

### 2.4. Voluntary Step Execution Test

Dynamic balance was assessed using the “voluntary step execution test” [14] that measures the ability of an individual to react to a disturbance and reestablish balance. The participants were positioned standing on parallel feet on a force platform (AMTI, Model OR 6-7, USA), sampling at 1 kHz, and looking at a fixed point at eye level 3 meters away from them. They were requested to perform a step forward as fast as they could immediately after a small tapping was provided manually by the experimenter at the heel (posterior aspect of the calcaneus bone prominence) of the dominant segment that was positioned in a backward position. A trial was considered valid when the participant maintained the balance after taking a step forward. After five trials of practice, three trials were recorded and the average was considered for further analysis. The force-time data allowed determining the duration of four movement phases: (1) step initiation, (2) preparation phase, (3) swing phase, and (4) step total time. The definition of these phases can be found elsewhere [14].

### 2.5. Functional Capacity Tests

Participants performed a battery of functional tests, which included the 6 minutes walking test (6MWT) that evaluates walking ability; the sit and reach test that evaluates lower back flexibility; the 8-foot up and go test that evaluates agility and balance; and the 30 s chair stand test that evaluates lower limbs muscle strength. Procedures for these tests have been described elsewhere [16].

### 2.6. Gait Analysis

Data were collected using the Plug-in-gait sacrum model of lower limbs, in which the union of the joints centers was used to determine the body segments and construct the biomechanical model for further analysis [17]. Six cameras (MX-13, Peak Vicon, UK), sampling at 100 Hz, identified the markers. The coordinates of these markers were filtered (quantic spline) and used to obtain 3D movement. The participants walked ten consecutive times in a self-selected speed on a walkway (8.0 m long; 1.2 m wide). One gait cycle (two consecutive heel ground contacts of the right limb) of each walking trial was captured when participants entered into the calibrated area (3 m long; 1.5 m wide; 1.7 m height). Then, three valid gait cycles were time normalized (0–100%) and averaged (ensemble average for further analysis). The following gait variables were determined: stride length (m), gait cycle duration (s), cadence (stride·s^−1^), gait speed (m·s^−1^), toe clearance (m), heel contact speed (m·s^−1^), swing duration (%), stance duration (%), and double support duration (*s*).

### 2.7. Maximal Dynamic Strength Test

Before starting the training period, the SG performed the one-repetition maximum (1RM) test to determine the initial training load of each participant [18]. The 1RM test was performed using the following weight stack machines, i.e., regular horizontal leg press, knee extensor, and flexor exercises (Nakagym). The participants performed two familiarization sessions, conducted in two consecutive days. In the first session, participants performed two sets of 10 repetitions using a light load (from 10 to 30 kg). In the second session, participants performed three submaximal repetitions to estimate 1RM and used it to the next session [19]. In the third session (1RM test), participants were allowed to warm up during 5 minutes in a treadmill and requested to execute one set of eight repetitions performed with a load that corresponded to 50% of the estimated 1RM. Then, after 2 minutes of resting, a second set of five repetitions was performed with a load that corresponded to 70% of the estimated 1RM. Finally, a maximal dynamic voluntary trial was performed with a maximal load that could be lifted one or two repetitions in a full range of motion [18]. When the participant completed more than two repetitions, the weight had been increased until a repetition failure, which corresponded not to lift the weight.

### 2.8. Exercise-Training Interventions

The SG and MG performed 36 training sessions on Mondays, Wednesdays, and Fridays, one-hour per session. Every SG session consisted of 10 min warming up, followed by 40 min of lower limbs and complementary exercises, and 10 min of cooling down. The lower limb SG program consisted of bilateral knee flexion and extension using sitting machines, horizontal leg press, hip adduction and abduction using sitting machines, standing plantar flexion in the step, and complementary exercises for upper limbs (bench-press, pulley, triceps, and biceps curl). Exercises were chosen to train all the lower limb muscles influencing the gait ability and the functional capacity. Participants completed three sets of eight repetitions of each exercise, with 50s resting between sets. In the first and the second training sessions, the workload was set at 60% of 1RM. In the following sessions, the workload was progressively increased adjusting the weight to perform eight maximal repetitions. The rating of perceived exertion during the SG sessions ranged from 12 to 14 on the Borg 6–20 Scale [20], similar to the previous study [21].

Multicomponent training sessions were divided into warm-up (5 min), gait, strength, balance and aerobic exercises (45-min), and stretching (10 min). During every week, two training sessions were focused on gait and strength exercises, whereas the remaining session was focused on balance and aerobic exercises [22]. Gait exercises consisted of 15–20 min of rapid changes in movement direction, e.g., fast zig-zag walking around cones. The progression was based on the complexity of task, such as bouncing the ball on the floor while zigzagging cones. Strength exercises consisted of 20–25 min of lower limb exercises using body weight (e.g., squat exercise) and/or elastic bands (e.g., knee flexion and extension sitting or standing; hip flexion and extension standing or lying) [23], performing 3 sets of 12 repetitions with 50s of resting between sets. The exercises were chosen considering all lower limb muscles that influence on gait ability and the functional capacity. The intensity of strength exercises was based on changing the resistance of the elastic bands. Participants started with an elastic band with lesser resistance and stiffness and progressively increased until higher resistance and stiffness [24]. The balance exercises included 15–20 minutes of static (e.g., standing with one foot) and dynamic exercises (e.g., walking on gymnasium court lines), and the progression was based on augmenting the instability of the supporting surface, e.g., standing or walking on a foam mattress. The aerobic exercise consisted of 20–25 min walking through the University campus. The intensity of aerobic training was controlled by the participants' perceived exertion that ranged from 12 to 14 on the Borg 6–20 Scale [20], i.e., moderate to vigorous exercise intensity [25].

### 2.9. Statistical Analysis

Shapiro–Wilk and Levene's tests confirmed data normality and homogeneity of variance of the variables, respectively. The dependent variables, which did not reveal significant differences between groups at pretraining evaluation, were compared with mixed model ANOVA having group (MG and SG) and time (pretraining and posttraining measurements) as fixed factors. In case of significant F-values, *post hoc* of Bonferroni was used for multicomparison purpose. The dependent variables of preparation phase and step total time of the voluntary step execution test differed between groups at pretraining evaluation and were compared with ANCOVA having the pretraining value as a covariable. The effect size was determined based on Cohen's *d* calculation for paired comparisons, pretraining and posttraining measurements for each group [26]. Reference values of *d* were (≤0.2) trivial, (0.21 to 0.49) small, (0.5 to 0.79) moderate, and (≥0.8) large effect. The level of significance was set at *p* < 0.05.

## 3. Results

### 3.1. Maximal Voluntary Isometric Contraction

There was interaction between training and time for hip flexion (*F*_(1,33)_ = 6,015; power = .663; *p*=0.020) and extension (*F*_(1,33)_ = 7,682; power = .767; *p*=0.009) and knee flexion (*F*_(1,33)_ = 12,881; power = .936; *p*=0.001) muscles (Table 1), in which the peak torque increased significantly from pretraining to posttraining in SG (20%, 36%, and 30%, respectively, to different muscles). In addition, the peak torque of hip flexors and extensors and knee flexors of the SG was significantly greater than that of the MG (23%, 28%, and 39%, respectively) at posttraining. There was main time effect for knee extensors (*p*=0.002) and plantar extensors (*p* < 0.01), with higher effect sizes for SG (*d*: −0.41 and −0.48). Moreover, there was main time effect for plantar flexors (*p*=0.017) with higher effect size for MG (*d* = −0.55).

There was training and time interaction for the rate of torque development of knee extension (*F*_(1,32)_ = 7,816; power = 0.774; *p*=0.009). This variable increased significantly from pretraining to posttraining for the SG (29%), while the MG did not change. In addition, the rate of torque development of knee extensors at posttraining for SG was significantly greater than MG (106%). There was neither interaction nor main effect for the rate of torque development of hip flexion (*F*_(1,33)_ = 3,231; *p*=0.081), hip extension (*F*_(1,32)_ = 0,409; *p*=0.527), knee flexor (*F*_(1,33)_ = 3,706; *p*=0.063), plantar flexor (*F*_(1,31)_ = 0,154; *p*=0.697), and dorsiflexor muscles (*F*_(1,32)_ = 0,249; *p*=0.621). These results are presented in Table 1.

### 3.2. Dynamic Balance

**Figure 2:**
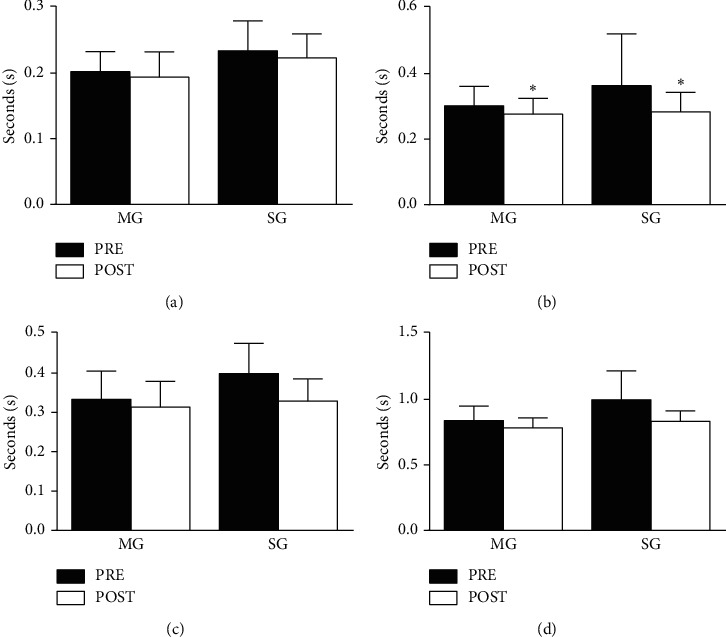
Voluntary step execution test for multicomponent and strength groups at pretraining and posttraining. ^*∗*^Main time effect and *p*=0.03. (a) Step initiation. (b) Swing phase. (c) Preparation phase. (d) Step total time.

There was no training and time interaction for step initiation (*F*_(1,28)_ = 0,041; *p*=0.840) and swing phase (*F*_(1,28)_ = 1,813; *p*=0.189). However, there was main time effect for swing phase duration (MG: *d* = .38; SG: *d* = .61) with time reduction to complete it. The ANCOVA did not present difference between groups in the preparation phase (*F*_(1,27)_ = 0,891; *p*=0.354) and step total time (*F*_(1,27)_ = 3,264; *p*=0.082) at posttraining. The results are presented in Figure 2.

### 3.3. Functional Capacity

**Figure 3:**
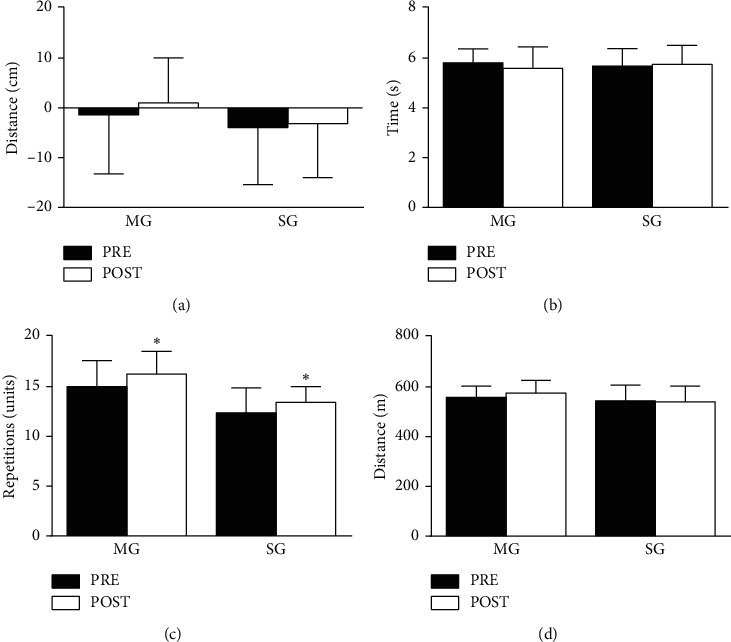
Functional capacity tests for multicomponent and strength groups at pretraining and posttraining. ^*∗*^Main time effect and *p*=0.02. (a) Sit and reach test. (b) 8 foot up and go test. (c) 30 s chair stand test. (d) 6MWT.

There was no training and time interaction for the 8-ft up and go test (*F*_(1,32)_ = 1,432; *p*=0.240), sit and reach (*F*_(1,32)_ = 0.368; *p*=0.548), 30-s chair stand (*F*_(1,22)_ = 0.056; *p*=0.815), and 6-min walking tests (*F*_(1,31)_ = 3.618; *p*=0.066), while there was main time effect for the 30-s chair stand test (MG: *d* = −0.54; SG: *d* = −0.48). The results are presented in Figure 3.

### 3.4. Gait Parameters

There was training and time interaction for stride length (*F*_(1,25)_ = 7.494; power = .749; *p*=0.011) and gait speed (*F*_(1,25)_ = 5.796; power = .638; *p*=0.024). These variables increased significantly for the MG (10% and 4%, respectively), while the SG did not change. There was no interaction for gait cycle (*F*_(1,25)_ = 5.796; *p*=0.024), cadence (*F*_(1,25)_ = 1.130; *p*=0.298), toe clearance (*F*_(1,25)_ = 0.686; *p*=0.415), heel contact speed (*F*_(1,25)_ = 1.787; *p*=0.193), limb stance (*F*_(1,25)_ = 2,910; *p*=0.100), swing duration (*F*_(1,25)_ = 3,192; *p*=0.086), gait cycle duration (*F*_(1,25)_ = 1.675; *p*=0.207), and double support duration (*F*_(1,25)_ = 0.524; *p*=0.476). However, there was performance improvement (main time effect) for toe clearance (MG: *d* = −0.66; SG: *d* = −0.31) and heel contact speed (MG: *d* = 1.07; SG: *d* = 0.25). The results are presented in Table 2.

## 4. Discussion

The aim of this study was to compare the effects of multicomponent and strength training programs on muscle function, dynamic balance, functional capacity, and gait pattern in older women. The results showed that SG had greater improvements in muscle function, whereas MG had greater improvements in gait ability. Moreover, both groups improved dynamic balance and functional capacity.

Peak torque improvements of hip flexion and extension and knee flexion of SG were greater than MG, similarly presented in the previous study [6]. Knee extension and plantar flexion and extension were improved, while the magnitude of the plantar flexors muscle improvements was greater to MG (*d* = −0.55) than to SG (*d* = −0.18). Multicomponent training may provide more stimuli to the plantar flexor muscles, due to walking and balance exercises included in the training program. The improvement of plantar flexor muscles is important to maintain balance after a postural disturbance in older adults, once during a reactive stepping these muscles are first activated before knee and hip muscles [3, 11, 27]. However, rate of torque development improvement was only found in the knee extensor muscles for SG. The difference between groups can be explained by more stimuli of strength training on rate of torque development [28] due to the adaptive changes in neuronal motor function, i.e., firing frequency and recruitment of motor units [9]. The absence of significant rate of torque development improvement for MG may be explained by the workload increment strategy (resistance and stiffness of the elastic bands) and with no focusing on fast movement execution, since strength and explosive-strength training have reported rate of torque development improvements [6, 28]. Thus, the strength training presents more ability to improve muscle function than multicomponent training in older women. Although, the multicomponent strategy may induce greater plantar flexor muscle strength gains, which is relevant to prevent falls in older adults.

The SG and MG presented improvements on the voluntary step test with greater magnitude of improvements for SG. The gains on peak torque of hip muscles and the rate of torque development of knee extensor muscles may explain the preparatory and swing phases improvements of SG, once these phases are dependent of neuromotor mechanisms, which are related to the buildup of muscle force and power [6, 14]. Peak torque of hip muscles is important to have a faster step execution, being a relevant skill to alter the base of support, preserve balance, and prevent falls in older adults [14]. Since multicomponent training had focused on daily living activities, with few exercises strengthen hip muscles, the improvements on balance may be minimized. Therefore, improvements on the voluntary step test after the training period indicate that strength training may be more effective to enhance dynamic balance than multicomponent, due to improvements on hip and knee muscle function.

Both groups improved the performance on 30 seconds chair stand test, with higher magnitude for MG. The improvement of functional capacity after a multicomponent training corroborates with the previous study [11]. It may be explained based on more exercise mechanical specificity of multicomponent than strength training [10]. Squat and walking exercises are more related to daily activities than isolated exercise movements in weight machines. Thus, multicomponent training may be more suitable type of exercise to improve functional capacity than strength training due to its relation to independence in daily-life activities in older adults [22].

Stride length and gait speed improved only for MG. A reduction as small as 0.1 m/s in habitual gait speed is associated with a 10% decrease in the ability to perform instrumental activities of daily living [29]. The improvements after multicomponent training may be explained by the agility and aerobic exercises performed in the training program. Moreover, toe clearance and heel contact speed improved for both groups, with higher effect sizes for MG. Such improvements for MG indicated that training programs with exercises involving more than one major muscle group and with similarity to activities of daily life may be better to improve toe clearance. It is an important parameter to the ability to clear obstacles, decrease the frequency of tripping, enhance propulsion during walking, and recovery during loss of balance [3]. Therefore, multicomponent training seems to be more effective to reduce parameters directly involved to fall risks in older adults than strength training.

In the present study, the absence of higher improvements in muscle function and functional capacity in MG after the training period may be explained based on the association of two factors. First, the exercise load increment may have underestimated the workload ability of the participants. Second, the participants could be considered physically active due to their high functional test scores at pretraining evaluation [30]. The previous study has reported higher improvement after strength and multicomponent training in participants with lower physical fitness level [31]. The high dropout rate during the experimental follow-up, that was higher than expected in MG, caused an unbalanced number of participants between the groups. Such unbalanced number may be a limitation considering the analysis of the present results. The participation of only older women restricts the application of the results to older men.

## 5. Conclusion

Strength training should be considered to increase muscle function and dynamic balance in older women, whereas multicomponent training should be considered to increase functional capacity and gait ability, which are variables strongly related to risk of falls.

## Figures and Tables

**Table 1:** Peak torque and rate of torque development around ankle, knee, and hip joints during maximal voluntary isometric contraction for multicomponent and strength groups at pretraining and posttraining.

	Multicomponent group		Strength group		*p* value
	Pre	Post	Pre	Post	
Peak torque					
Hip flexion (N.m)	47.2 (23.1)	47.1 (15.4)	48.3 (14.5)	58.0 (14.7)^ab^	**0.020**
Hip extension (N.m)	108.1 (72.3)	99.0 (45.8)	93.5 (40.9)	126.9 (37.9)^ab^	**0.009**
Knee flexion (N.m)	24.9 (9.3)	25.9 (8.9)	27.6 (8.3)	35.9 (8.3)^ab^	**0.001**
Knee extension (N.m)^*∗*^	58.5 (29.6)	69.6 (32.1)	81.3 (29.2)	93.9 (31.4)	0.842
Plantar flexion (N.m)^*∗*^	15.0 (6.1)	19.4 (8.5)	20.2 (8.7)	21.6 (6.5)	0.198
Plantar extension (N.m)^*∗*^	12.9 (5.4)	15.3 (5.3)	15.2 (6.0)	18.8 (7.8)	0.364

RTD					
Hip flexion (N.m.s^−1^)	0.27 (0.24)	0.21 (0.16)	0.24 (0.14)	0.27 (0.11)	0.081
Hip extension (N.m.s^−1^)	0.26 (0.25)	0.27 (0.45)	0.35 (0.22)	0.31 (0.14)	0.527
Knee flexion (N.m.s^−1^)	0.12 (0.07)	0.11 (0.07)	0.12 (0.05)	0.16 (0.08)	0.063
Knee extension (N.m.s^−1^)	0.16 (0.10)	0.15 (0.10)	0.24 (0.13)	0.31 (0.17)^ab^	**0.001**
Plantar flexion (N.m.s^−1^)	0.06 (0.06)	0.05 (0.04)	0.07 (0.05)	0.06 (0.03)	0.697
Plantar extension (N.m.s^−1^)	0.03 (0.01)	0.03 (0.01)	0.05 (0.02)	0.05 (0.02)	0.621

^*∗*^Main time effect *p* < 0.05; a: different from pretraining; b: different from multicomponent group at posttraining; RTD: rate of torque development.

**Table 2:** Gait parameters for multicomponent and strength groups at pretraining and posttraining.

	Multicomponent group		Strength group		*p* value
	Pre	Post	Pre	Post	
Stride length (m)	1.12 (0.11)	1.17 (0.12)^a^	1.17 (0.14)	1.16 (0.14)	**0.011**
Gait cycle (s)	1.08 (0.06)	1.03 (0.04)	1.06 (0.07)	1.05 (0.09)	0.207
Cadence (Stride.s−^1^)	0.93 (0.06)	097 (0.04)	0.95 (0.06)	0.96 (0.07)	0.298
Gait speed (m.s−^1^)	1.03 (0.11)	1.13 (0.12)^a^	1.11 (0.18)	1.11 (0.19)	**0.024**
Toe clearance (cm)^*∗*^	3.91 (0.55)	4.32 (0.67)	3.94 (0.68)	4.13 (0.50)	0.415
Heel contact (m.s−^1^)^*∗*^	0.87 (0.22)	0.65 (0.20)	0.84 (0.32)	0.76 (0.29)	0.193
Limb stance (%)	59 (1)	57 (1)	59 (2)	59 (2)	0.100
Swing phase (%)	41 (2)	43 (1)	41 (2)	41 (2)	0.086
Double support (s)	0.10 (0.03)	0.10(0.02)	0.10 (0.02)	0.09 (0.02)	0.476

^*∗*^Main time effect *p* < 0.04; a: different from pretraining.

## Data Availability

The data used to support the findings of this study are available from the corresponding author upon request.
